# Capture - recapture based study on the completeness of smear positive pulmonary tuberculosis reporting in southwest Iran during 2016

**DOI:** 10.1186/s12889-021-12398-w

**Published:** 2021-12-23

**Authors:** Homayoun Amiri, Mohammad Javad Mohammadi, Seyed Mohammad Alavi, Shokrolah Salmanzadeh, Fatemeh Hematnia, Mahnaz Azar, Heydar Rahmatiasl

**Affiliations:** 1 Master of Epidemiology, Infectious and Tropical Diseases Research Center, Health Research Institute, Ahvaz Jundishapur University of Medical Sciences, Ahvaz, Iran; 2 Infectious and Tropical Diseases Research Center, Health Research Institute, Ahvaz Jundishapur University of Medical Sciences, Ahvaz, Iran; 3 Department of Environmental Health Engineering, School of Public Health AND Air Pollution and Respiratory Diseases Research Center, Ahvaz Jundishapur University of Medical Sciences, Ahvaz, Iran; 4 Professor of Infectious and Tropical Diseases, Infectious and Tropical Diseases Research Center, Health Research Institute, Ahvaz Jundishapur University of Medical Sciences, Ahvaz, Iran; 5 General Practitioner, Infectious and Tropical Diseases Research Center, Health Research Institute, Ahvaz Jundishapur University of Medical Sciences, Ahvaz, Iran; 6 Expert in Laboratory Sciences, Infectious and Tropical Diseases Research Center, Health Research Institute, Ahvaz Jundishapur University of Medical Sciences, Ahvaz, Iran; 7 Master of Health Education, Infectious and Tropical Diseases Research Center, Health Research Institute, Ahvaz Jundishapur University of Medical Sciences, Ahvaz, Iran

**Keywords:** Tuberculosis, Completeness of reporting, Capture recapture, Iran

## Abstract

**Background:**

Tuberculosis (TB) is one of the ten leading causes of death in infectious diseases and one of the ten leading causes of death in the world. For any TB control program, a valid surveillance is essential. In order to assess the status of the assessment, the quality of the record and the completeness of reporting should be assessed. The purpose of this study was to investigate the completeness of smear positive pulmonary tuberculosis reporting in Ahvaz, south west of Iran.

**Methods:**

This cross-sectional study was conducted in 2016 in Ahvaz, southwest Iran. The study was conducted through a three-source Capture recapture method by collecting laboratory, hospital, physician prescription data; including patient referral to the health care center, prescriptions of patients receiving anti-tuberculosis drugs and prescriptions of medical TB diagnostic laboratories, and laboratory prescriptions. Percentage, mean and standard deviation were used to describe the variables. Data analysis was performed using log-linear model in Rcapture package R software.

**Results:**

Generally, 134 new cases of smear-positive pulmonary tuberculosis patients were reported through three sources from urban and rural regions during 2016. Pulmonary tuberculosis was reported through three sources from urban and rural regions during 2016. The most common age group was 25 to 44 years and 79.1% of the patient were man. The overall prevalence of new cases of smear-positive pulmonary tuberculosis was in persons that lived urban areas (97.8%). The completeness of reporting the disease estimated by log-linear model was 87.5% and the incidence rate was estimated to be 11.8 disease per 100,000 persons. Completeness of reporting of laboratory, hospital and physician resources were 79%, 30% and 16.3%, respectively.

**Conclusions:**

The present study shows the necessity of evaluating the quality, completeness and linkage between data. Linking between data sources can improve the accuracy and completeness of TB surveillance.

## Background

The presence of *Mycobacterium Tuberculosis* in at the level of human societies is a serious public health concern. Tuberculosis is the most common cause of death from single-factor infectious diseases and one of the ten leading causes of death in the world [1, 2]. One of the most ways for Tuberculosis (TB) control is understanding of TB epidemiology [2]. Observation of infectious diseases as well as tuberculosis is most important for public health [3, 4]. In the context of the end TB strategy, is vital national assessment [5].

According to the report of the world health organization (WHO) (2020), the incidence of tuberculosis was estimated about 14 per 100,000 in Iran for 2020, while 11 per 100,000 have been recorded and reported in the same year [6]. It seems that progress in Preventive and diagnostic measures and advanced assessment and treatment of the TB disease in global, regional and country levels should be seriously taken into consideration [6].

Underreporting is an important issue in assessing the status of the surveillance for communicable diseases, which leads to underestimation of the burden of disease and as a result can make disease control difficult [7, 8]. Based on strategies WHO post-2015 emphasize the importance of universal access and enhance the effectiveness of the TB observation in order to minimize this disease [9]. In addition, complete reporting of tuberculosis patients and subsequent timely treatment plays an important role in disease control [7]. In this regard, in order to properly interpret the status of tuberculosis and its trends, the quality of disease registration and reporting should be assessed [3]. There are several ways to evaluate the completeness of reporting, such as the capture recapture method [10]. The world health organization uses this method to estimate the incidence of tuberculosis in some countries [11]. In addition, numerous studies have evaluated the completeness of reporting tuberculosis by capture recapture method over a specific period in specific geographical areas [12]. According to several conducted study in different region in the world such as Greek, Baleric Iceland, Iraq, Yemen, South Africa and Liverpool, England completeness of reporting tuberculosis was investigated using three-source capture recapture method [13–18]. In addition to record-linkage of two or more tuberculosis registers, capture-recapture studies have been performed in the field of tuberculosis observation [19].

Due to the up-growing population, increasing urbanization, presence of people in closed environments most of the day, the emergence of emerging and re-emerging diseases, proximity of Iran to the underdeveloped countries such as Afghanistan, Pakistan and Iraq, illegal entry of illegal immigrants and considering that Khuzestan province is one of the provinces with a high prevalence of tuberculosis infection in the country, it shows the necessity of early treatment and diagnosis of tuberculosis in order to reduce the rate of incidence and mortality.

The purpose of this study was to investigate the completeness of smear positive pulmonary tuberculosis reporting in Ahvaz, south west of Iran during 2016.

## Methods

### Type and area of study

This cross-sectional study was conducted in the geographical area of Ahvaz city in Khuzestan province of southwest Iran in year 2016. In this study, 643 people a TB test was requested by the health care services (sputum smear microscopy, chest X-ray or culture), diagnosis by register identical and record similar demographic data and information on (provisional) diagnosis, treatment and referral of patients, with additional verification of health care services diagnosis information. There were 664 prescriptions for tuberculosis medication, but none contained two or more drugs (The prescriptions in which one of the TB drugs was mentioned because of some TB drugs are commonly used to treat other infectious diseases, including malaria and meningitis). A total of 134 new smear positive pulmonary TB cases selected from 8 urban districts of the city. The data gathering was based on diagnostic criteria for pulmonary TB patients. The population of individuals diagnosed with mycobacterium tuberculosis sputum specimen were enrolled in the study. In this study our cases were selected according to WHO and Ministry of Health of Iran criteria such as clinical findings, a smear sample, a chest radiography (C-X ray) and culture [20–22].

**Fig. 1:**
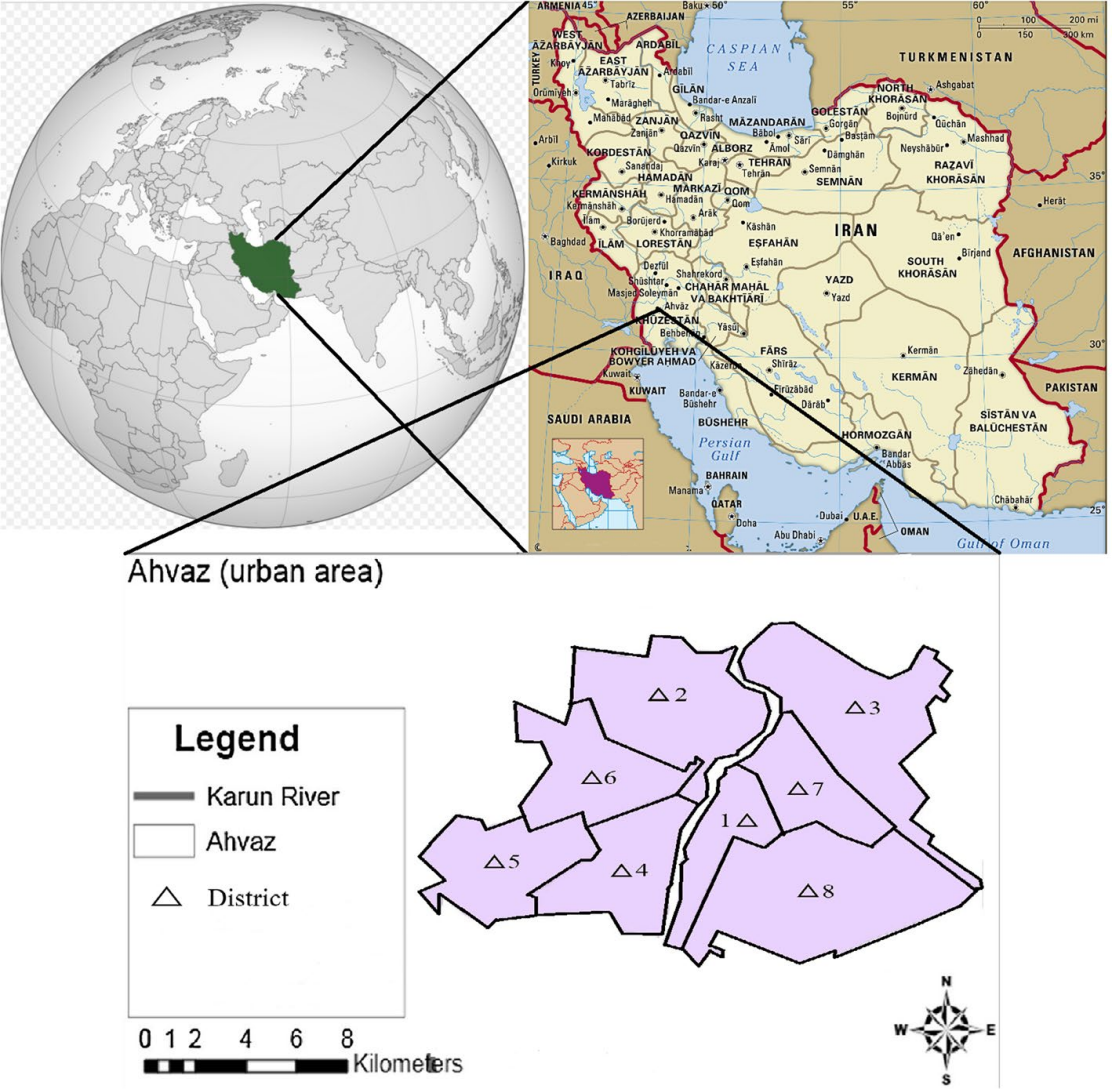
Geographical map of the site study (Ahvaz in southwest of Iran)

Ahvaz with 1.2 million inhabitants approximately, an area of 63,238 km^2^, is one of the largest metropolitan’s city in the Iran and Middle East. Ahvaz is located in in southwest of Iran. Ahvaz is the capital Khuzestan province [23, 24]. Location of Ahvaz is presented in Fig. 1.

### Inclusion and Exclusion criteria

Inclusion criteria for entering this study were all a positive test response to Mycobacterium tuberculosis, age, method of laboratory diagnosis and newness of the patient in the city.

Exclusion criteria for the study were false-positive cases, cases with insufficient identifiers for perfect matching, none contained two or more TB drugs in prescriptions for tuberculosis medication because of some TB drugs are commonly used to treat other infectious diseases; (including malaria and meningitis) and verification of assumed true-positive tuberculosis patients among non-culture-confirmed tuberculosis cases [19].

### Tuberculosis surveillance

Tuberculosis care system for notification of cases in the Islamic Republic of Iran is integrated with the structure of the health care system. In such a way, within all levels of health care provision (health house, health base, health centers, policlinic, clinics and hospitals) in case suspected person of having pulmonary tuberculosis is detected, a smear sample is prepared from the person and sent to laboratories. The selected will be sent. If the test results or a chest radiography (C-X ray) are positive in favor of tuberculosis, the patient is introduced to the tuberculosis treatment unit of the city health center and is treated standard.

### Data collection

In this study, we used to three-source log-linear model capture-recapture studies on infectious disease incidence [25]. We determined number of test requested based on urban divisions of city by regions, population of each region and number of laboratories. First a list of laboratories that perform TB diagnostic tests was obtained by referring to the University of Medical Sciences. Then, by referring to these laboratories in the public and private sectors and reviewing the records of patients’ test results, the national code for patients with pulmonary tuberculosis was received. Individuals diagnosed with Mycobacterium tuberculosis sputum specimen were included in the study. The data of these patients were collected from central laboratories of East and West health centers of Ahvaz and private laboratories throughout the city, hospital (for collect information from this source, we referred to the hospitals of Ahvaz city. Patients admitted to public and private hospitals in Ahvaz city with diagnosis of TB positive based on an International Code for Diseases (ICD-9) for active tuberculosis (ICD-9 codes 010–018) [3]. The information of these patients was extracted through hospital information system and the physician reporting (The possibility of using more sources of information, such as prescription drugs, etc.) [26].

Physician reporting data source were considered both physicians’ reports in the form of introducing patients to the city’s health centers and both prescription and laboratory prescriptions. Doctors’ reports were obtained in the form of introducing patients with tuberculosis to health centers, in coordination and referring to comprehensive urban and rural health centers in Ahvaz. Also, by coordinating and referring to the insurance organizations (Social Security Insurance and Iranian Health Insurance), we collected the information of pharmaceutical and laboratory prescriptions registered in these two organizations. In general, insurance companies are connected to all pharmacies and laboratories through an integrated electronic network and receive any request for medicine and testing for the insured person. Some patients may have referred to specialist physicians in neighboring provinces for treatment, diagnosis and the inability to access these patients’ prescriptions were among the most important limitations of the study.

To gather information from this source, both physician reporting in the form of patient referrals to health centers in the city and pharmaceutical and laboratory prescriptions were considered. In the study of the drug prescription, the condition of inclusion in the study was the prescription of complete drug administration for the treatment of the disease. In the laboratory version, all the copies that requested Acid-Fast Bacillus (AFB) or Polymerase Chain Reaction (PCR) testing were also examined. The list of people who had been asked for a test was obtained from the insurer organizations. There were 664 prescriptions for tuberculosis medication, but none contained two or more drugs. For 643 people a test was requested in Ahvaz (the number 643 refers to the number of people suspected of having TB for whom a doctor has requested a diagnostic test, and no medicine will be prescribed until the test results are known). The two numbers do not necessarily have to be equal because of some TB drugs are commonly used to treat other infectious diseases, including malaria and meningitis. All medications prescribed for the treatment of patients were also reviewed. In all sources the inclusion criteria for entering the study were a positive test response to Mycobacterium tuberculosis and newness of the patient.

According to reported WHO-approved was necessary to allow for the capture of cases diagnosed using rapid diagnostic tests (such as Xpert MTB/RIF) by reporting system [27]. This current revision requests the reporting of all new and relapse case notifications by age and sex [27].

### Communication between data

At first, each source item was individually imported into Excel. Then duplicates in each source were identified and deleted. Patients’ information was merged to find commonality between the three lists. Patients’ characteristics including national code and patient identification number were considered. By sorting the patients in different order was done based on their national code or patient identification number. Using the sort command and comparing each case with those recorded in another source, commonalities were identified.

### Statistical analysis

Descriptive statistical measures including mean, standard deviation and percent used to describe the data. The estimated prevalence rates presented with 95% confidence interval (95% CI). The analyses were carried out with log-linear model in Rcapture package R software. The structural source models potential interdependencies of the registers and heterogeneity of the population. Explore this method as a tool for periodic evaluation of the WHO tuberculosis control strategy in a resource-limited setting.

### Capture recapture analysis

**Fig. 2:**
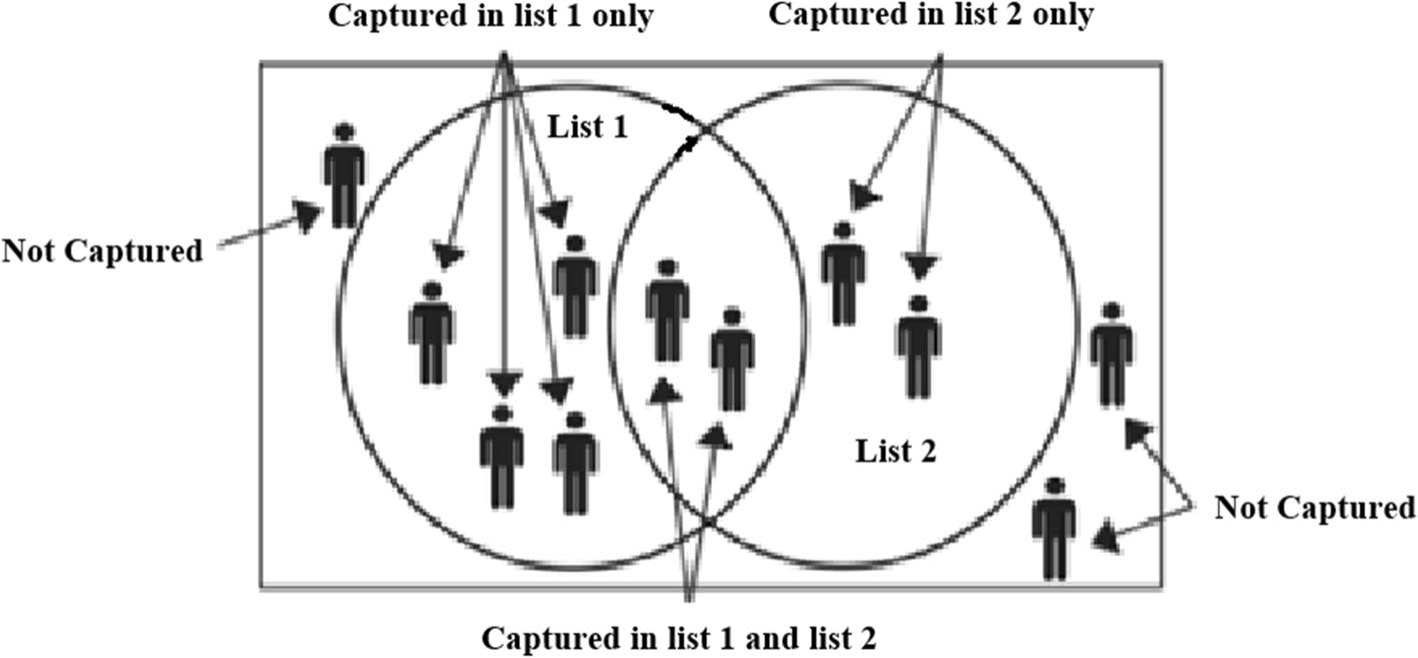
Illustration Capture-Recapture method

The study was conducted using three sources Capture-Recapture method. This method estimates the total number of expected cases based on the number of unregistered cases [7]. Data analysis was performed using log-linear model in Rcapture package R software. To estimate the number of individuals not recorded in any of the three sources, eight different log-linear model were fitted to the available data, and the frequency was estimated using each of these models. The log-linear model takes into account the dependence and heterogeneity of the resources in the calculation and is very robust when there are multiple sources [28]. Logarithmic test of likelihood ratio (${G}^{2}$), degree of freedom, Akaike information criterion (AIC) and Bayesian information criterion (BIC) were used to check the fit of the models to the available data and to select the best model. AIC and BIC were tested by Likelihood ratio tests and lower values indicate better fit [28]. The most common criterion for selecting the appropriate model was AIC [29]. In cases where the sample size is low, the other two indices, in particular BIC, give better results [28]. Illustration Capture-Recapture method showed in Fig. 2.

## Results

**Fig. 3:**
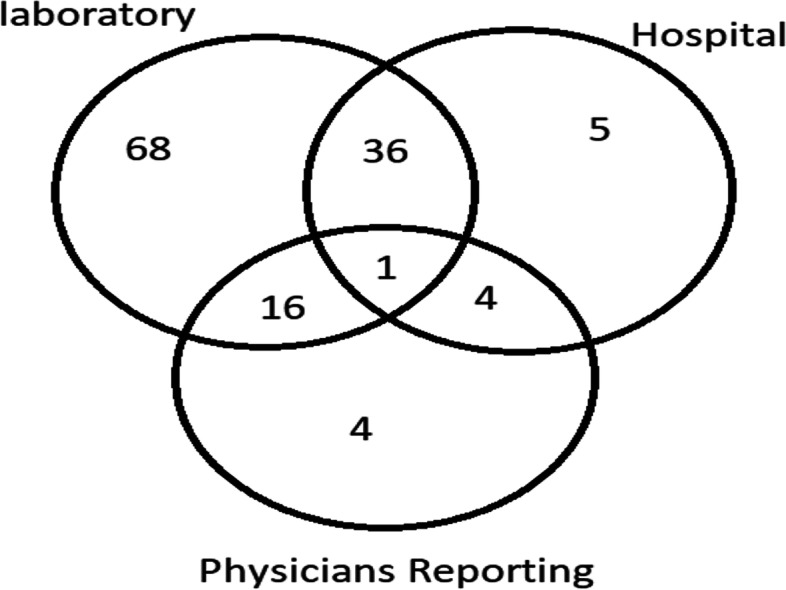
Venn diagram for showed duplicates of smear positive pulmonary tuberculosis cases in Ahvaz city in 2016

A total of 134 new smear positive pulmonary TB cases selected from 8 urban districts of the city. The data gathering was based on the peoples of diagnostic criteria for pulmonary TB patients. The population of individuals diagnosed with mycobacterium tuberculosis sputum specimen were enrolled in the study. After comparing cases in three sources and eliminating common duplicates between sources-counting once in common - a total of 134 new smear positive pulmonary TB cases were recorded in the laboratory, the hospital and the physician reporting source in 2016. The laboratory source recorded 121 cases, the hospital source 46 cases and the physician reporting source 25 cases. The total number of new cases reported in the three sources were 192 cases that 58 new cases were duplicate smear-positive TB results that should be delete. We used a Venn diagram for showed duplicates of smear-positive pulmonary tuberculosis cases in three sources and the common cases (Fig. 3**)**.

The incidence rate of smear-positive pulmonary tuberculosis in Ahvaz based on information surveillance data, the data of the present study, and linear logarithm estimation were 9.8, 10.3, and 11.8 diseases per 100,000 people, respectively.

According to the result of this study, the number of new smear positive pulmonary TB cases in man and woman were 106 (79.1%) and 28 (20.9%), respectively (Table 1). The ratio of man to woman was 3.7. The result showed that the highest age group was in the age group of 25-44 years with 50.7%, followed by the age group of 45-64 years with 20.8% (Table 1). Based on result of this study, 97.8% were urban and 2.2% lived in rural areas. Table 1 shows the demographic characteristics of patients recorded by the laboratory, the hospital and the physician reporting source.

**Table 1:** descriptive of demographic information of patients from three sources of smear positive pulmonary tuberculosis registry in 2016

Basic information		Number	Percentage
Sex	Man	106	79.1
	Woman	28	20.9
Age	15**>**	0	0
	15-24	18	13.5
	25-44	68	50.7
	45-65	28	20.8
	65<	20	15
Living area	urban	131	97.8
	rural	3	2.2

Table 2 shows the various three-source log-linear model capture-recapture studies of infectious disease incidence and completeness of notification with the number of patients observed and their frequency counts, the objective of the study, the data sources used and the selected log-linear model. Three-source analysis was performed using log-linear model in R software. information criterion estimated for smear positive pulmonary TB was 59 (Table 2). According to this model, the number of smear-positive pulmonary TB cases not recorded in any of the sources was estimated to be 19. As a result, the total number of smear-positive pulmonary tuberculosis cases was estimated to be 153 (95% confidence interval: 134-142) in 2016. Table 2 showed that the completeness of reporting rate of smear-positive pulmonary tuberculosis for (laboratory, hospital and physician reporting source with 121, 46 and 25 cases were estimated to be 79%, 30% and 16.3%, respectively) was 87.5%. In the present study, the Bayesian Information Criterion (BIC) statistic was used to select a model that fits the data better, and a model that included independent effect between hospital source, laboratory and physician reporting.

**Table 2:** Information of log-linear model fitted to smear positive pulmonary TB data in 2016

Model	Info Fit	Df	BIC	Estimate the total number of cases	95% confidence interval	Completeness of reporting in percentage	
L.H.R	ok	3	59	153	142-164	87.5	
LR.H	ok	61	145	139-151	92.4		
LH.LR.HR	Warning	0	62	135	-	-	
HR.L	ok	2	62	151	144-158	88.7	
LR.HR	ok	63	143	137-149	93.7		
LH.R	ok	2	64	155	143-167	86.4	
LH.LR	ok	1	64	139	134-144	96.4	
LH.HR	ok	1	67	151	140-162	88.7	

L (Lab), H (Hospital), R (Physician Reporting), Info fit (Indicates the presence or absence of a model error(, (Degree of freedom) DF, (Bayesian Information Criterion) BIC

A comparison between the frequency and incidence of disease in surveillance data was done and data collected in the present study and linear log estimation was presented in Table 3. Using linear logarithmic model, the incidence of disease was estimated to be 11.8 disease per 100,000 persons in 2016 (Table 3).

**Table 3:** Comparison between frequency and incidence of smear positive pulmonary tuberculosis in Ahvaz

	Frequency of TB	Disease per 100,000 people
Surveillance data	128	9.8
The data of the present study	134	10.3
Linear logarithm estimation	153	11.8

## Discussion

Our findings illustrate that laboratory, hospital, physician prescription (patient referral to the health center, patients receiving anti-tuberculosis drugs and medical TB diagnostic laboratories) data should be check and register for all TB cases. The study illustrates that the highest number of patients with new smear positive TB was observed in man (25-44 years).

Based on the results of this study, the completeness of reporting smear-positive pulmonary tuberculosis in Ahvaz city using the data of three sources of the hospital, laboratory, and physician reporting was 87.5%.

Also, result from this study demonstrated that the incidence of disease was estimated to be eleven and eight-tenths of a patient per one hundred thousand people based on the various three-source log-linear model capture-recapture model.

This underreporting leads to lack of timely and standard treatment (Dots) of TB patients, so improving the care system (TB) of tuberculosis by improving timely reporting of cases of positive pulmonary tuberculosis smear and subsequent timely treatment of patients leads to a decrease in cases of TB, death from disease and resistant TB will be treated.

Besides, timely reporting of tuberculosis cases and subsequent contact tracing will play an important role in preventing the spread of the disease.

Investigation of the relationship between smear-positive pulmonary tuberculosis parameters of gyms and density of *Mycobacterium Tuberculosis* needs to gather information such as age, sex, and living area. For human diseases, capture-recapture analysis has predominantly been applied to estimate the prevalence, incidence, or completeness of registers of specific groups of diseases, often diseases with a chronic character as mentioned earlier. Apparently the characteristics of most of these diseases, their patients and their registers best fulfil criteria for feasibility of capture-recapture studies as well as validity of the underlying assumptions [19].

The strategy to end tuberculosis by 2030 pursues goals such as a 90% reduction in mortality and an 80% reduction in disease incidence [30]. To achieve these goals, diagnosis and treatment of patients has an important role to play, which also requires an optimal surveillance [26]. The incidence rate of smear-positive pulmonary tuberculosis in Ahvaz based on information surveillance data, the data of the present study, and linear logarithm estimation were 9.8, 10.3, and 11.8 diseases per 100,000 people, respectively.

Also, result this study estimated to frequency of TB according to information surveillance data, the data of the present study, and linear logarithm estimation were 128, 134, and 153 persons, respectively. The number of differences in cases was six patients. Data about 4 (66%) cases of this was not registered in the surveillance system because of they were dead before the disease was diagnosed.

Edginton et al. was also mentioned this point in their study [31]. This can have an impact the indicators of assessing the status of the surveillance, 3% in reducing the success rate of treatment and equally in increasing Mortality rate from the disease. Using the log-linear model, a model that includes the independent effect of each source, the number of cases not recorded in any of the sources was estimated to be 19, which is consistent with the results of the Dunbar et al. Study in South Africa [17]. This study shows that, the completeness of reporting smear-positive pulmonary tuberculosis was 87.5%, which is similar to the results of studies in France and Romania [12, 32] and the World Health Organization’s Executive Task Force on Tuberculosis Control, which provides for the detection of at least 70% of positive smear tuberculosis cases [17]. The highest percentage of completeness of reporting (79%) was related to laboratory data, which was consistent with Vanina Guerrier’s study in France, Cojocaru’s study in Romania, and Ibarz-Pavon’s study in Greece [12, 13, 32].

According to a 2016 World Health Organization report, the estimated incidence of all forms of tuberculosis in Iran is estimated at 16 per one hundred thousand [33]and Therefore, considering the ratio between different forms of tuberculosis, the incidence estimated by this organization is lower than the rate calculated in this study. It should be noted, however, that this estimate is for the entire population of Iran, while the incidence and prevalence of tuberculosis are high in the marginal areas of Iran including, Khuzestan province [34]. The assumptions of Capture-recapture studies, such as population closure, the possibility of finding commonality between sources, the independence of resources from each other, and the dependence of the catch on the specificity of the individuals at the time of these studies, should be considered [17]. In this study, due to the use of Excel software and sort data by name, surname and, national code and manual review of all records, the default breach is that it is limited to find commonality between resources. The study also included a population closure assumption and included only patients who resided in the study area, but because this city is the center of the province, some patients may have mentioned their relatives’ address at the time of hospitalization and, so were included in the study. The default breach of catch dependency regarding individuals’ characteristics is limited due to the widespread use of primary health care at the county level and the free diagnosis and treatment of tuberculosis.

In Capture-recapture studies, by including the interaction between different sources, the effect of dependence (positive or negative) between the sources can be taken into account in the estimates and, bias due to the lack of default independence of resources can be largely eliminated [35].

In this study, the elimination of duplicates prevented overestimation and, since only those with laboratory confirmation were included in the study, the accurate default of diagnosis was considered and, no false positives remained in the data.

In another study, Smit et al. estimate the completeness of notification of incident tuberculosis cases in the Netherlands [3]. They reported that between 1499 tuberculosis patients which were identified, of whom 1298 were notified, resulting in an observed under-notification of 13·4% [3]. Also, prediction by Log-linear capture–recapture analysis initially a total number of 2053 (95% CI 1871–2443) tuberculosis cases [3]. The result of this study showed that the total number of smear-positive pulmonary tuberculosis cases was estimated to be 153 (95% confidence interval: 134-142). This difference in the number of tuberculosis patients can be because of population, economic status of the society, level of awareness, and culture of the society and geographical conditions.

Huseynova et al. in Iraq studied tuberculosis burden and reporting in resource-limited countries [15]. Based on the result of this study, a total of 1985 TB cases registered 1677 patients (observed completeness 84%). They investigated total number of TB cases was 2460 (95%CI 2381–2553), with identified TB cases representing 81% (95%CI 69–89) [15]. Huseynova et al. administrated that TB surveillance needs to be strengthened to reduce under-reporting. This administrated is the same identical as our study.

In Egypt by Bassili et al. evaluation of tuberculosis case detection rate in resource-limited countries [5]. According to result this study CDR of NTP surveillance and completeness of case ascertainment after record linkage was respectively 55% (95%CI 46–68) and 62% (95%CI 52–77). They stated that sputum smear-positive TB cases, these proportions were 66% (95%CI 55–75) and 72% (95%CI 60–82), respectively [5].

In the three-source capture analysis, data collected from each source should be more than 15% of the total catch and have sufficient overlap [17]. In the present study, disease cases from laboratory, hospital, and physician reporting sources dedicated were 90%, 34%, and 18%, respectively.

The results of this study indicate that under-reporting of smear-positive tuberculosis cases in Ahvaz was about 12.4%. Based on our result study, the cases with positive smear pulmonary TB which had laboratory confirmation them were 3.9%.

The highest overlap was between the laboratory and hospital sources and, the lowest overlap was between hospital sources and physician reporting which, were inconsistent with the study results by Dunbar et al. [17]. It is suggested to report the disease from the hospital and laboratory level using electronic systems to eliminate the challenge of not registering patients in the TB treatment system and given strengthening the approach of electronic medical records in recent years. In addition, the cases of tuberculosis admitted to the hospital can be seasonally extracted and compared with reported cases by examining the hospital registration system. Continuous evaluation of the disease care system using, the capture-recapture method is also recommended.

### Limitations

This study did not cover cultural factors and economical patient information. One of the main limitations of this study was discussed only the factors that influence compliance with TB.

Also, referred to specialist physicians in neighboring provinces for diagnosis, treatment and the inability to access these patients’ prescriptions were another of the limitation of this study. Observed findings showed that another significant limitation of this study includes the limitations of the population of individuals.

The limitations to capture-recapture studies estimating tuberculosis incidence or prevalence depend on the violation of the underlying assumptions.

## Conclusions

Our findings showed that the maximum source reported new smear-positive pulmonary TB cases by the laboratory with recorded 121 new cases. In summary, we, found that the overall prevalence rate of smear-positive tuberculosis cases in Ahvaz was low, especially in women. In this study, we investigated the capture-recapture based study on the completeness of smear-positive pulmonary tuberculosis reporting in Ahvaz city during 2016. The average ratio of mans to women was 3.7.

The result showed that the improve the cure rate and treatment of the smear-positive population patients it is necessary and can reduce mortality significantly. Our results suggest that for estimating pulmonary TB incidence and completeness of notification, independent and parsimonious three-source log-linear capture–recapture models are preferable.

The relevant medical departments should strengthen the supervision and intervention of the TB treatment process, strengthen TB-related essential knowledge propaganda, raise awareness of TB patients, and give financial and policy support to farmers and herders in remote areas to improve anti-TB treatment adherence.

## Data Availability

Upon request, we can offer onsite access to external researchers to the data analyzed at Ahvaz Jundishapur University of Medical Sciences, Ahvaz, Iran. To do so, Dr. Homayoun Amiri should. be contacted.

## Abbreviations

TB: Tuberculosis
AFB: Acid-Fast Bacillus Testing
PCR: Polymerase Chain Reaction testing
AIC: Akaike Information Criterion
BIC: Bayesian Information Criterion
WHO: World Health Organization
